# Croatian population and clinical epidemiologic studies (1995-2011); the use of ABC score and gradual progress of cardiosurgical model in developing countries

**DOI:** 10.1186/1749-8090-8-S1-O117

**Published:** 2013-09-11

**Authors:** I Malčić, D Dilber, D Šarić, H Kniewald, D Bartoniček, D Anić, D Belina, A Hodalin, S Dorner, N Čače, V Metličić, K Markičević, M Sršen-Krstulović

**Affiliations:** 1 Clinical Hospital Centre Zagreb, Department of Paediatric Cardiology and Department of Cardiac Surgery, Zagreb, Croatia

## Summary

The treatment of the children with congenital heart disease (CHD) is not the same in different parts of the world. In Europe, a patway for international cooperation in the field of care for children with CHD is not yet well established, and higly dependent on national structures and cooperation. We established the Register of CHD in Croatia using the priciples of EUROCAT, with the aim to study the distribution of CHD and outcome analysis. The prevalence of CHD in two different periods (January 1. 1995- December 31. 2000 and January 1. 2002- Dezember 31. 2007) was 0.8 and 0.72%. By employing the widely used and accepted methodologies of case mix complexity adjustment in congenital cardiac surgery, together with collecting data in EUROCAT Registry and European Association for Cardio-Thoracic Surgery Database (EACTS), we tried to study surveillance of children in Croatia and to evaluate our performance. All children born in Croatia between January first 2003 and December 31. 2013 with diagnosis of CHD were included. Case mix complexity adjustement of cardiac operation is performed by using the Aristotel basic complexity score. During study 1034 patients were operated and 1278 operatins performed. Among 641 operations performed in Croatia 25 children died during or as a result of operations, that gives 3.9% of early miortality. The mean complexity for cardiac procedures peroformed in Croatia is 5.9. More complex operations were perfomed in foreigen centres with ABC score more between 6-15 and early mortality score less than 5%. During the last 4 years of the study, the number of operations performed in Croatia showed linear increase: 55, 78, 121 and 126 operations were performed in 2008, 2009, 2010 an 2011, rrespectively.

## Conclusion

Further initiatives have to be done, to improve the services provided on a national level and also to develop cooperation between neghboring small countries.

